# Regulator of G-protein signalling 2 mRNA is differentially expressed in mammary epithelial subpopulations and over-expressed in the majority of breast cancers

**DOI:** 10.1186/bcr1834

**Published:** 2007-12-08

**Authors:** Matthew J Smalley, Marjan Iravani, Maria Leao, Anita Grigoriadis, Howard Kendrick, Tim Dexter, Kerry Fenwick, Joseph L Regan, Kara Britt, Sarah McDonald, Christopher J Lord, Alan MacKay, Alan Ashworth

**Affiliations:** 1 Breakthrough Breast Cancer Research Centre, The Institute of Cancer Research, Fulham Road, London SW3 6JB, UK; 2 The Ludwig Institute for Cancer Research, Riding House Street, London W1W 7BS, UK

## Abstract

**Introduction:**

To understand which signalling pathways become deregulated in breast cancer, it is necessary to identify functionally significant gene expression patterns in the stem, progenitor, transit amplifying and differentiated cells of the mammary epithelium. We have previously used the markers 33A10, CD24 and Sca-1 to identify mouse mammary epithelial cell subpopulations. We now investigate the relationship between cells expressing these markers and use gene expression microarray analysis to identify genes differentially expressed in the cell populations.

**Methods:**

Freshly isolated primary mouse mammary epithelial cells were separated on the basis of staining with the 33A10 antibody and an α-Sca-1 antibody. The populations identified were profiled using gene expression microarray analysis. Gene expression patterns were confirmed on normal mouse and human mammary epithelial subpopulations and were examined in a panel of breast cancer samples and cell lines.

**Results:**

Analysis of the separated populations demonstrated that Sca-1^- ^33A10^High ^stained cells were estrogen receptor α (Esr1)^- ^luminal epithelial cells, whereas Sca-1^+ ^33A10^Low/- ^stained cells were a mix of nonepithelial cells and Esr1^+ ^epithelial cells. Analysis of the gene expression data identified the gene *Rgs2 *(regulator of G-protein signalling 2) as being highly expressed in the Sca-1^- ^33A10^Low/- ^population, which included myoepithelial/basal cells. RGS2 has previously been described as a regulator of angiotensin II receptor signalling. Gene expression analysis by quantitative real-time RT-PCR of cells separated on the basis of CD24 and Sca-1 expression confirmed that *Rgs2 *was more highly expressed in mouse myoepithelial/basal mammary cells than luminal cells. This expression pattern was conserved in normal human breast cells. Functional analysis demonstrated RGS2 to be a modulator of oxytocin receptor signalling. The potential significance of RGS2 expression in breast cancer was demonstrated by semi-quantitative RT-PCR analysis, data mining and quantitative real-time RT-PCR approaches, which showed that *RGS2 *was expressed in the majority of solid breast cancers at much higher levels than in normal human mammary cells.

**Conclusion:**

Molecular analysis of prospectively isolated mammary epithelial cells identified RGS2 as a modulator of oxytocin receptor signalling, which is highly expressed in the myoepithelial cells. The *RGS2 *gene, but not the oxytocin receptor, was also shown to be over-expressed in the majority of breast cancers, identifying the product of this gene, or the pathway(s) it regulates, as potentially significant therapeutic targets.

## Introduction

Signal transduction pathways are commonly dysregulated in breast cancer. Estrogen receptor signalling is the best characterised aberrant signalling pathway in the disease [1], but others include the human epidermal growth factor receptor (HER)2, epidermal growth factor receptor (EGFR), prolactin receptor and oxytocin receptor pathways [2-7]. A striking feature of these receptors and their downstream pathways is that they are important determinants of the growth, development and function of the normal breast epithelium [3,4,8,9]. It is likely that the competence of mammary epithelial cells to respond to these signalling pathways in normal development leads to selective pressure to recruit these molecules into the process of tumourigenesis. Understanding the molecular mechanisms that underlie normal cell growth and functional differentiation in the normal mammary gland is therefore critical to developing new therapeutic approaches that target these signalling pathways. A key step in developing such an understanding must be knowledge of genes expressed in the different cellular compartments of the mammary epithelium and their relationship to the function(s) of that compartment.

The adult mouse mammary epithelium consists of a network of ducts together with (in the pregnant/lactating gland) milk-producing alveoli, the latter being equivalent to the terminal ductal lobulo-alveolar units in human [10]. Both of these structures consist of two basic cell layers: an inner luminal epithelial layer and an outer basal epithelial layer. Luminal cells line the ducts, form the differentiated milk-secreting cells in the alveoli, and are the principal target for estrogen and prolactin. The basal layer is mainly composed of myoepithelial cells, which contract in response to oxytocin released during lactation to force milk from the alveoli down the ducts to the nipple. The basal cell layer also contains the stem cell compartment, which maintains the epithelium [11-14].

We have previously used a variety of cell surface markers to isolate and characterize these populations. These have included a mouse luminal epithelial milk fat globule membrane antigen (recognized by the 33A10 antibody [15,16]) to isolate mouse mammary luminal epithelial cells [17,18]; CD24 to isolate both basal/myoepithelial and luminal epithelial cells [12,14]; and Sca-1 and Prominin-1 (the mouse homologue of CD133), which both recognize estrogen receptor-α positive (Esr1^+^) luminal epithelial cells [14]. We have shown that the basal epithelial compartment is enriched for epithelial stem cell activity [12,14] and confirmed that this activity can be further purified on the basis of high CD49f expression [13,14]. In our previous studies, however, we confined molecular analysis of these populations to characterizing their function on the basis of expression of genes with known or predicted cell-type specific distributions.

The aim of the present study, therefore, was to identify genes that have not previously been characterized as being differentially expressed in mammary epithelial cell populations, with particular focus on genes with dysregulated expression in breast cancer. As a result of this analysis we have identified the G-protein coupled receptor (GPCR) regulator *RGS2 *(which encodes the regulator of G-protein signalling 2 [RGS2]) as being differentially expressed in the various breast epithelial populations. Furthermore, this gene is over-expressed in the majority of breast cancers, making it a potential new target for the development of therapeutics.

## Materials and methods

### Antibodies

Anti-mouse Sca-1-PE antibody (clone D7; used at 0.1 μg/ml), mouse adsorbed anti-rat IgG-FITC (used at 1 μg/ml) and nonspecific rat IgG control antibodies were obtained from Southern Biotechnology (Cambridge Bioscience, Cambridge, UK). 33A10 rat IgG antibody supernatant (used undiluted) was a kind gift from Professor A Sonnenberg (Netherlands Cancer Institute, Amsterdam, The Netherlands). Rat anti-mouse CD45-PE-Cy5 and CD45-PE-Cy7 (clone 30-F11; used at 0.25 μg/ml) and anti-CD24-FITC (clone M1/69; used at 0.5 μg/mL) were obtained from BD Biosciences (Oxford, UK). Anti-CD24-PE-Cy5 (clone M1/69; used at 0.25 μg/ml) was obtained from Insight Biotechnology (London, UK).

### Plasmids

The *RGS2 *coding sequence without 5' or 3' untranslated regions was isolated from an IMAGE clone (3681138; Geneservice, Cambridge, UK) in pDNR-LIB by PCR using the primers 5'-GGCTCGAGGCCGCCACCATGCAAAGTGCTATGTTCTTG-3' and 5'-GGCTCGAGTCATGTAGCATGAGGCTC-3'. These primers added a Kozak consensus sequence to the 5' end and *Xho*I restriction enzyme recognition sites to both ends of the amplified sequence. The PCR product was cloned using the pCR-TOPO-XL kit (Invitrogen, Paisley, UK) and then subcloned from this vector into pcDNA3.1- (Invitrogen) by *Xho*I digest. *Hind*III restriction digests indicated plasmids with the RGS2 sequence in the correct orientation (shown by excision of a 510 base pair fragment). Plasmids with the *RGS2 *insert in the correct orientation were sequence verified.

### Preparation and flow cytometric separation of single mammary cell suspensions

Mammary epithelial organoids were harvested from fourth mammary fat pads of virgin female FVB mice aged 10 to 12 weeks and processed to single cells as previously described [12]. For anti-Sca-1/33A10/anti-CD24 sorting, cell suspensions (at 10^6 ^cells/ml) were stained as detailed in Table 1. Staining and sorting with anti-CD24-FITC and anti-Sca-1-PE for isolation of mouse mammary basal/myoepithelial, Esr1^+ ^luminal and Esr1^- ^luminal cells was carried out as described previously [14]. Analysis and exclusion of dead cells, CD45^+ ^cells and nonsingle cells was carried out as described previously [12]. Nonspecific IgG controls were used for compensation and to set sort gates. Flow cytometry data was analysed using FlowJo [19].

**Table 1:** Multiple staining protocols for flow cytometric analysis of α-Sca-1/33A10/α-CD45 and α-Sca-1/33A10/α-CD45/α-CD24 stained cells.

Sample	Antibody/antibodies			ToPRO-3 or DAPI
		
	First incubation	Second incubation	Third incubation	
Nonspecific staining control	Rat immunoglobulin	α-rat-FITC	IgG-PE IgG-PE-Cy5 ^2^IgG-PE-Cy7	No
ToPRO-3 or DAPI control	None	N/A	N/A	Yes
33A10 control	33A10	Anti-rat-FITC	N/A	No
Sca-1 control	α-Sca-1-PE	N/A	N/A	No
CD45 control	^a^α-CD45-PE-Cy5 or ^b^α-CD45-PE-Cy7	N/A	N/A	No
^b^CD24 control	^b^α-CD24-PE-Cy5	N/A	N/A	No
Experimental sample	33A10	α-rat-FITC	α-Sca-1-PE ^a^α-CD45-PE-Cy5 or ^b^α-CD45-PE-Cy7 ^b^α-CD24-PE-Cy5	Yes

^a^Anti-CD45-PE-Cy5 was used for the four-colour protocol not including CD24. ^b^Anti-CD45-PE-Cy7, anti-CD24-PE-Cy5 and the IgG-PE-Cy7 isotype control were only used in the five-colour protocol including CD24 detection. 'Fluorescence minus one (FMO)' controls based on the staining combination used for the experimental sample but in which one antibody was left out and replaced with its isotype control were also used to set sort gates correctly. For simplicity, these have not been shown. Live/Dead cell exclusion used either ToPRO-3 or DAPI (4,6-diamidino-2-phenylindole dihydrochloride).

### Cell culture

Hs578T cells (Sigma, Poole, Dorset, UK) were cultured in Dulbecco's modified Eagle's medium (Invitrogen) with 10% (vol/vol) foetal calf serum (FCS; PAA Laboratories, Somerset, UK) and 10 μg/mL insulin (Sigma). Primary mouse fibroblasts isolated during the preplating procedure in the mammary cell harvest were cultured in Dulbecco's modified Eagle's medium/10% (vol/vol) FCS for up to 2 weeks before RNA isolation.

### cDNA microarray gene expression analysis on freshly isolated mammary epithelial cells

Cells were freshly isolated from mammary tissue, with no intervening culture period, and sorted into sterile screw-cap eppendorf tubes. After sorting, cells were pelleted by spinning in a benchtop centrifuge at 700 *g *for 5 minutes. Phosphate-buffered saline (PBS) supernatant was aspirated and cell pellets resuspended in 800 μl Trizol reagent (Invitrogen). Where necessary, multiple pellets of the same population were pooled in the 800 μl volume. Cultures of primary mouse mammary fibroblasts were washed in PBS and then scraped into Trizol. Samples were stored at -80°C until required for RNA extraction. Total RNA was extracted according to the manufacturers' instructions from two independent Sca-1^+ ^33A10^Low/- ^samples, two independent Sca-1^- ^33A10^Low/- ^samples, three independent Sca-1^- ^33A10^High ^samples, and four independent mammary fibroblast samples. Quality and quantity of the total RNA was assessed using an RNA 6000 nano chip on the Agilent Bioanalyzer (Agilent Technologies UK Limited, Stockport, Cheshire, UK). One hundred nanograms of total RNA was amplified using the AminoAllyl MessageAmp aRNA kit (Ambion, Huntingdon, UK), in accordance with the manufacturer's protocol.

Reference RNA was generated from a pool of RNAs extracted from three mammary cell preparations harvested and sorted to exclude CD45^+ ^cells only. This total RNA pool was amplified using the same technique. Five micrograms of amino allyl aRNA from both test and reference RNA were coupled with either Cy3 or Cy5 fluorochromes (GE Healthcare, Buckinghamshire, UK) and purified with an AminoAllyl MessageAmp aRNA kit (Ambion).

Dye-labelled test and reference aRNA were co-hybridised on to an in-house (Breakthrough Breast Cancer Centre) cDNA mouse microarray containing 13,825 features (NIA 15K Mouse cDNA clone set) [20,21]. Slides were incubated in a 42°C hybridization oven for 16 hours. Washes performed once in 2× sodium chloride/citrate (SSC) and 0.1% (w/v) SDS for 15 minutes, followed by three washes in 0.1 × SSC and 0.1% (weight/vol) SDS for 10 minutes each, and two final washes in 0.1 × SSC for 2 minutes each time. All washes were performed at 65°C. Slides were spin dried before scanning.

### Microarray data analysis

Each hybridization was performed in duplicate as a dye swap hybridization. Slides were scanned using an Axon 4000B scanner (Axon Instruments, Burlingame, CA, USA) and images were analysed using Genepix Pro 5.1 software (Axon Instruments). Aberrant or distorted spots were removed from analysis. Expression measurements were obtained after log_2 _transformation of raw intensities and print-tip lowess normalization within each array [22]. Differential gene expression was determined by using the limma package [23] in the R 2.1.1 environment [24] and BioConductor 1.6 [25]. Differentially expressed genes were ranked according to their *P *value and M ratio, and genes with a false discovery predication of *P *value < 0.05 were included for further studies [26]. A full list of significantly over-expressed and under-expressed annotated genes from the four populations is given in Additional files 1 to 4. A combined list of all the genes from Additional files 1 to 4, allowing comparisons of the *P *values across the populations, is given in Additional file 5. The complete datasets, together with unpublished analyses, have been submitted according to MIAME (Minimum Information About a Microarray Experiment) guidelines [27] to the public data repository ArrayExpress [28] with accession number E-MEXP-423.

### Data mining

The gene expression data from normal luminal epithelial cells, myoepithelial cells and epithelial enriched primary breast cancers were obtained from the reported study by Grigoriadis and coworkers [29]. The expression pattern for human *RGS2 *was extracted using the Unigene ID Hs.78944 as an identifier.

### Semi-quantitative RT-PCR analysis of cell lines and breast tumour samples

Total RNA (10 μg) from primary samples and cell lines derived from both normal breast and breast cancers was used for each 40 μl reverse transcription reaction. Samples included the following: a normal, immortalized but nontransformed human myoepithelial cell line 1089M (derived from primary myoepithelial cells purified from human reduction mammoplasty tissue and immortalized with SV40 large T-antigen and hTERT, and then subjected to a second round of myoepithelial specific purification; a kind gift from Dr Mike Allen, Queen Mary's School of Medicine and Dentistry, London); a pool of RNA from 10 freshly isolated primary breast luminal epithelial cell samples [29]; immortalized but nontransformed human luminal cell lines 1089L, HB4A [30], 226L33, 226L39 (normal human breast luminal cells immortalized with temperature-sensitive SV40 large T-antigen; a kind gift from Professor Parmjit Jat, Ludwig Institute for Cancer Research) and HBL100 [31]; normal human breast derived endothelial cells and fibroblasts [32]; a panel of ESR1^+^, ERBB2^+^, myoepithelial origin and non-estrogen responsive cell lines [33]; and 56 primary breast cancers, of which 20 had been purified to remove F19 antigen-expressing desmoplastic fibroblasts (termed F19^- ^breast cancers) [29]. Unless otherwise stated, samples were a kind gift of Professor Mike O'Hare, Ludwig Institute for Cancer Research. To determine the expression in this panel of *RGS2*, *OXTR *(encoding oxytocin receptor) and a control house keeping gene *B2M *(encoding β_2_-microglobulin), 10 μl of 1/50 diluted cDNA was used per 30 μl RT-PCR with primers for *RGS2 *(5'-CGAGGAGAAGCGAGAAAAGA-3' and 5'-TTCCTCAGGAGAAGGCTTGA-3'), *OXTR *(5'-TTCTTCGTGCAGATGTGGAG-3' and 5'-GGACGAGTTGCTCTTTTTGC-3'), or *B2M *(5'-ACTCTGCTTAGAATTTGGGG-3' and 5'-CCACAACCATGCCTTACTTT-3'). Absence of contaminating genomic DNA was confirmed by analysis of samples in an Agilent Bioanalyser. The *RGS2 *and *OXTR *primers were designed such that they spanned one or more intron-exon boundaries and would give bands of the expected size (150 base pairs for *RGS2 *and 233 base pairs for *OXTR*) only when amplifying from cDNA not from genomic DNA.

RT-PCR was performed by using the Applied Biosystems AmpliTaq Gold (Applied Biosystems, Warrington, UK), with 32 (*RGS2*), 36 (*OXTR*) or 25 (*B2M*) cycles each consisting of 30 seconds at 94°C, 30 seconds at 60°C, and 45 seconds at 72°C. PCR products were visualized on 2% (weight/vol) agarose E-Gels 96 Gels (Invitrogen). An IMAGE clone of *RGS2 *(3681138) and cDNA made from RNA isolated from Hs578T cells, which express a functional oxytocin receptor [34], were used as positive controls for the *RGS2 *and *OXTR *PCRs, respectively.

### Quantitative PCR analysis

Quantitative real time PCR (qPCR) reactions were carried out as described previously [14] to determine fold changes in expression of *Esr1 *(estrogen receptor-α) and *Prlr *(prolactin receptor) [14] in mammary epithelial cell subpopulations compared with a leucocyte-depleted, bulk mammary cell (CD45^-^) comparator sample. For qPCR assays on mouse *Rgs2 *(Unigene ID Mm.28262; Taqman Assays on Demand reference Mm00501385_m1) or human *RGS2 *(Unigene ID Hs. 78944; Taqman Assays on Demand reference Hs00180054_m1), fold changes in expression were compared with mouse or human mammary fibroblasts. In all cases, *GAPDH *(Taqman Assays on Demand reference for mouse Mm99999915_g1, for human Hs00266705_g1) was used as an internal control. Data were expressed as mean fold changes across samples together with 95% confidence intervals. Significance was determined by comparing confidence interval overlaps [35].

Analysis of normal primary human cells was carried out on two pools of RNA from freshly isolated normal myoepithelial cells and two pools of RNA from freshly isolated normal luminal cells (a kind gift of Professor Mike O'Hare). Each cell pool was derived from at least 10 separate isolates of normal primary human breast cells. Each of the myoepithelial pools was analyzed in duplicate, using two separate cDNA syntheses. One luminal pool was analysed in duplicate and one in triplicate with separate cDNA syntheses. Analysis of cell lines and tumour samples was carried out on a selection of the samples described above.

### Transfections

For small interfering (si)RNA analysis, Hs578T cells were seeded at 1.4 × 10^5 ^cells per well in six-well plates (Falcon; BD Biosciences) in antibiotic-free medium. The following day, cells were transfected with either an siControl#1 (siCON) siRNA (D-001210-01; Dharmacon, Perbio Science Belgium, Erembodegem, Belgium) or an siRNA targeting human *RGS2 *(Hs_RGS2_1_HP siRNA; Qiagen, Crawley, West Sussex, UK; siRGS2) using Lipofectamine reagent (Invitrogen), in accordance with the manufacturer's instructions. The response of the cells to oxytocin stimulation (see below) was measured 48 hours later. As a transfection control, additional cells were transfected with an siRNA targeting the *PLK1 *(polo-like kinase 1) gene (hs_PLK1_6_HP; Qiagen), which is lethal (Lord C, Ashworth A, unpublished data). To confirm gene silencing, qPCR analysis of *RGS2 *expression levels in siCON versus siRGS2 transfected wells was carried out.

To establish cell lines stably over-expressing RGS2, Hs578T cells were transfected with *Pvu*I-linearized pcDNA3.1-RGS2, empty pcDNA3.1- or mock transfected, using Lipofectamine 2000 (Invitrogen), in accordance with the manufacturer's instructions. After 48 hours, cells were selected with 0.5 mg/ml Genetecin (Invitrogen), sufficient to select completely against nontransfected cells. The mock transfected cells all died, but multiple colonies survived in the transfected cultures. The cell lines were maintained as polyclonal populations, under Genetecin selection, to minimize potential variation due to plasmid integration effects.

### Oxytocin receptor activity assays

To assess oxytocin receptor signalling to the downstream p44/42 mitogen-activated protein kinase (MAPK) pathway [36], control and transfected cells in six-well plates were serum and insulin starved for 2 hours and then stimulated with either 1 × 10^-7 ^mol/l (*RGS2 *silencing) or 5 × 10^-7 ^mol/l (*RGS2 *over-expression) oxytocin (Sigma) for varying times up to 2 hours. At each time point, medium was removed, the cells were washed with cold PBS and then scraped into 0.5 ml cold lysis buffer (50 mmol/l Tris [pH 7.4], 5 mmol/l EDTA, 150 mmol/l NaCl, 1% Igepal, 2 mmol/l DTT, 1:100 protease inhibitor cocktail, 1:100 phosphatase inhibitor cocktail 1, and 1:100 phosphatase inhibitor cocktail 2; all reagents from Sigma). Samples were lysed on ice for 20 minutes and then spun at 25000 *g *in a benchtop centrifuge for 10 minutes at 4°C. Pelleted insoluble material was discarded and Bradford protein assays carried out on the supernatants. Supernatant samples were mixed with 2 × SDS loading buffer and analysed by SDS-PAGE on 10% polyacrylamide gels and Western blotting. Sample loading was normalised using the results of the Bradford assays. Blots were probed with antibodies against Phospho-p44/42 MAPK (Thr202/Tyr204; #9101; Cell Signalling Technology, NEB, Hitchin, Herts, UK) and total p44/42 MAPK (#9102; Cell Signalling Technology), in accordance with the manufacturer's instructions. A Typhoon Phosphoimager (GE Healthcare, Buckinghamshire, UK) was used to quantify the intensity of the phosphorylated and total bands. The ratios of the corresponding bands was determined and then the percentage of p44/42 phosphorylation at each timepoint was compared with time 0 (0 minutes). Statistical analysis on the p44/42 phosphorylation levels was carried out using a χ^2 ^method, testing whether the observed level of phosphorylation in the Hs578T-RGS2 cells or siRNA transfected cells treated with oxytocin differed significantly from the expected level of phosphorylation (in the control samples treated with oxytocin) across the timepoints.

To assess oxytocin signalling-mediated elevation of intracellular calcium levels, a calcium flux assay based on the Fluo-3/Fura Red system was used [37]. In brief, cells were trypsinized and re-suspended at 5 × 10^5^/ml in room temperature phenol-red-free Leibowitz L15 medium/10% (vol/vol) FCS plus 0.1 mmol/l sulfinpyrazone (Sigma). Fluo-3-AM and Fura Red-AM (Invitrogen) were then added to final concentrations of 1 and 2 μmol/l, respectively. Cells were incubated at room temperature for 30 minutes then washed and resuspended in room temperature phenol-red-free L15/10% FCS plus 0.1 mmol/l sulfinpyrazone plus 1:10,000 DAPI (4,6-diamidino-2-phenylindole dihydrochloride). After a further 30 minutes at room temperature the samples were analyzed on a Becton Dickenson LSRII flow cytometric analyser (Becton Dickenson, Oxford, UK). Dead cells and doublets were gated out of the analysis, as described previously [12], and the ratio of Fluo-3/Fura Red fluorescence was plotted against time. This ratio increases with increasing [Ca^2+^]_i_. The fluorescence ratio for each sample was analysed using FlowJo for 60 seconds before addition of 5 × 10^-7 ^mol/l oxytocin, to obtain a baseline value, and then for 150 seconds afterward. To compare samples, mean fluorescence ratios for the baseline readings and for 25 second intervals after stimulus addition were plotted.

### Ethical approval

Harvest of animal tissue for cell separation was carried out under Schedule 1 of the 1986 Animals (Scientific Procedures) Act. Informed consent to use human material for scientific research was obtained.

## Results

### 33A10 and α-Sca-1 staining identifies mammary cell populations with distinct gene expression patterns

**Figure 1:**
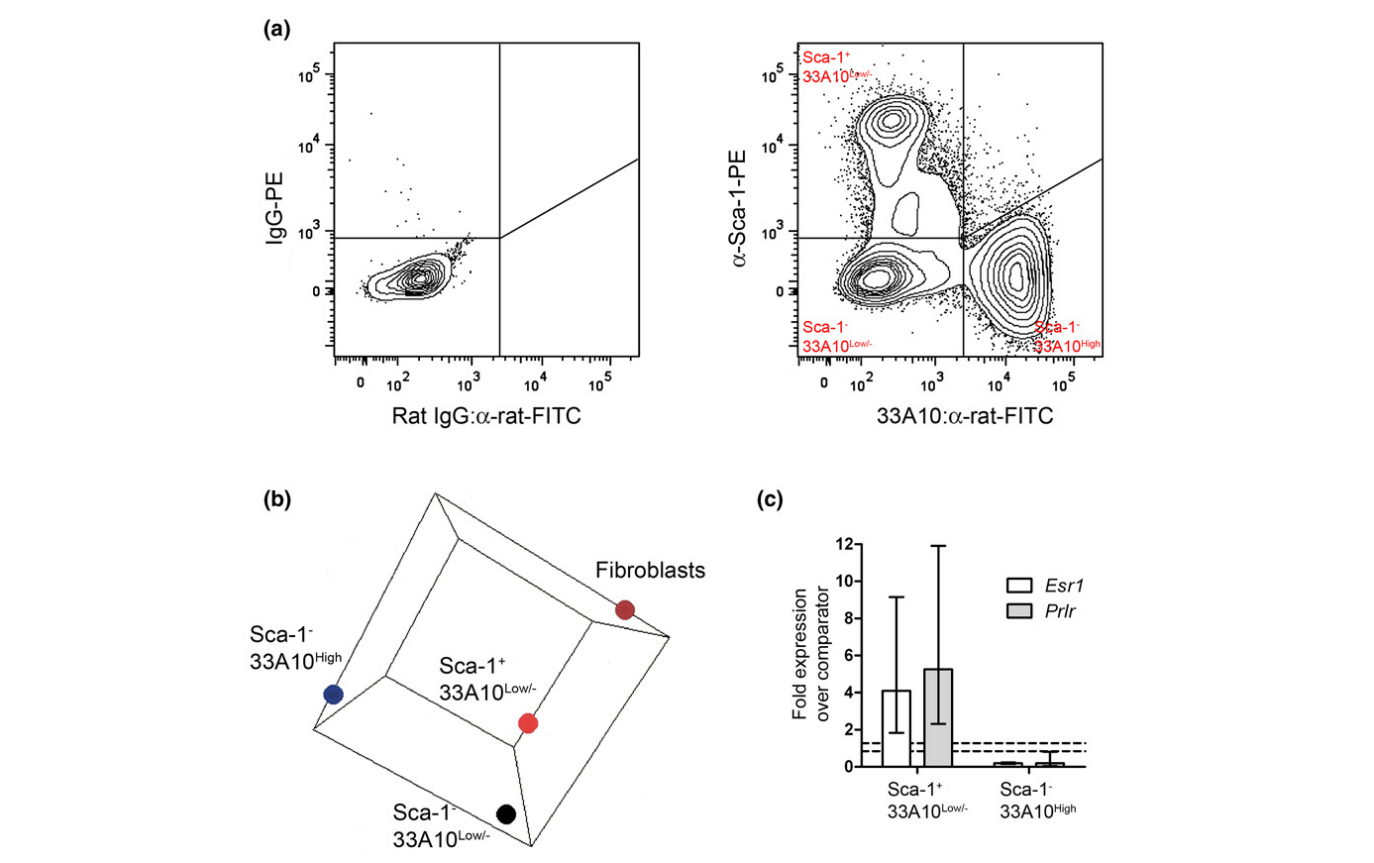
Isolation and characterisation of mammary epithelial cell subpopulations. **(a) **Flow cytometric staining profiles (dead and CD45^+ ^cells excluded) of anti-Sca-1 and 33A10 stained, freshly isolated mouse mammary cell preparations together with nonspecific IgG-stained control. **(b) **Graphical space representation of principal component analysis of mammary fibroblasts, Sca-1^+ ^33A10^Low/-^, Sca-1^- ^33A10^High ^and Sca-1^- ^33A10^Low/- ^cells. **(c) **Mean fold differences ± 95% confidence limits in RNA abundance measured by quantitative real-time PCR for estrogen receptor (*Esr1*) and prolactin receptor (*Prlr*) transcripts in Sca-1^+^33A10^Low/- ^(*n *= 5 samples) and Sca-1^- ^33A10^High ^(*n *= 3 samples) mammary subpopulations compared with bulk mammary cell preparations depleted for CD45^+ ^cells (comparator; *n *= 3 samples). The dotted lines indicate the 95% confidence limits of the comparator sample. All samples show a significant difference to the comparator (**P < 0.01) [35].

We previously characterized the antibody 33A10 as an exclusive marker of mouse mammary epithelial luminal cells [17,18] and have demonstrated that Sca-1 expression is a marker of estrogen receptor-α expressing cells within the luminal epithelial compartment [14]. Therefore, to isolate mammary luminal epithelial cell subpopulations for gene expression analysis, freshly harvested primary mammary cell suspensions were stained with 33A10, anti-Sca-1 and anti-CD45 antibodies, and separated by flow cytometry. Dead cells and CD45^+ ^cells were excluded from the analysis. A typical flow cytometry profile is shown in Figure 1a. Three main cell populations were identified: Sca-1^+ ^33A10^Low/- ^(14.7 ± 4.8%), Sca-1^- ^33A10^High ^(44.3 ± 8.0%) and Sca-1^- ^33A10^Low/- ^(25.4 ± 6.3%; *n *= 5 independent sorts). Surprisingly, given that 33A10 is a luminal epithelial cell marker and α-Sca-1 stains Esr1^+ ^luminal epithelial cells, no substantial Sca-1^+ ^33A10^High ^population was observed.

Isolated Sca-1^+ ^33A10^Low/-^, Sca-1^- ^33A10^High ^and Sca-1^- ^33A10^Low/- ^cells were used to generate gene expression profiles for each of these subpopulations, compared with a reference sample consisting of pooled bulk mammary CD45^- ^cell preparations. In addition, gene expression patterns in mammary fibroblasts, isolated by differential plating during the cell preparation procedure, were also compared with the same reference sample. Additional files 1 to 4 list the well annotated differentially expressed genes significantly increased and decreased in the four populations compared with bulk CD45^+^-depleted mammary cells according to limma (false discovery rate method) analysis. Additional file 5 compares gene expression in all four populations.

To understand the relationship between the four cell populations, principal component analysis was carried out on the four datasets (Figure 1b). This analysis showed that the Sca-1^- ^33A10^High ^and fibroblast populations were most distinct, whereas the Sca-1^+ ^33A10^Low/- ^and the Sca-1^- ^33A10^Low/- ^populations had the greatest similarity. However, none of the populations clustered together, confirming that the flow cytometric separation did indeed isolate three largely separate populations, which themselves are all different to the mammary fibroblasts.

To confirm that the Sca-1^+ ^33A10^Low/- ^population included the Sca-1^+ ^hormone receptor-expressing luminal epithelial cells that we previously identified [14], qPCR for the prolactin receptor and estrogen receptor-α (*Prlr *and *Esr1*) was carried out on Sca-1^+ ^33A10^Low/- ^and Sca-1^- ^33A10^High ^cells. The results (Figure 1c) demonstrated that Sca-1^+ ^33A10^Low/- ^cells were indeed enriched for *Prlr *and *Esr1 *expression, whereas Sca-1^- ^33A10^High ^luminal epithelial cells were depleted for expression of these genes.

To characterize the biological processes that may be occurring within each cell type, and therefore to better understand their function, Gene Ontology analysis of the biological processes to which each enriched gene contributes was carried out (Additional file 6). This analysis showed that significant processes elevated in Sca-1^- ^33A10^High ^cells involved ion and lipid metabolism and transport processes. This was not unexpected for cells of the luminal mammary epithelium. Interestingly, however, phagocytosis was also found to be an important biological process in this cell population. The Sca-1^- ^33A10^Low/- ^cells, Sca-1^+ ^33A10^Low/- ^cells and the mammary fibroblasts showed a number of similarities, for instance cell adhesion, proteolysis and peptidolysis, which is consistent with the presence of nonepithelial cells in all three populations. However, the Sca-1^- ^33A10^Low/- ^cells also had a distinct functional speciality involving genes encoding proteins that are associated with smooth muscle contraction. This was consistent with this population containing myoepithelial cells.

### 33A10^High ^luminal epithelial cells are cognate with CD24^High ^Sca-1^- ^luminal epithelial cells

The data suggested that both the Sca-1^- ^33A10^Low/- ^and Sca-1^+ ^33A10^Low/- ^populations were a mixture of epithelial and nonepithelial cell types, whereas the Sca-1^- ^33A10^High ^population was a relatively pure population of Esr1^- ^and Prlr^- ^luminal epithelial cells. Only a few genes were found to be upregulated in this population compared with the reference, and only at modest levels, probably as a consequence of the reference population used being itself mainly composed of Sca-1^- ^33A10^High ^cells. Nevertheless, the genes that were upregulated (for example, that encoding lactotransferrin) or downregulated (for example, *Prlr*) were consistent with the Sca-1^-^/33A10^High ^cells being cognate with the CD24^High ^Sca-1^- ^Prominin-1^- ^Esr1^- ^mouse mammary luminal epithelial cells that we recently identified [14]. This contrasted with previous observations that 33A10 stained all mouse luminal epithelial cells [15,17,18].

**Figure 2:**
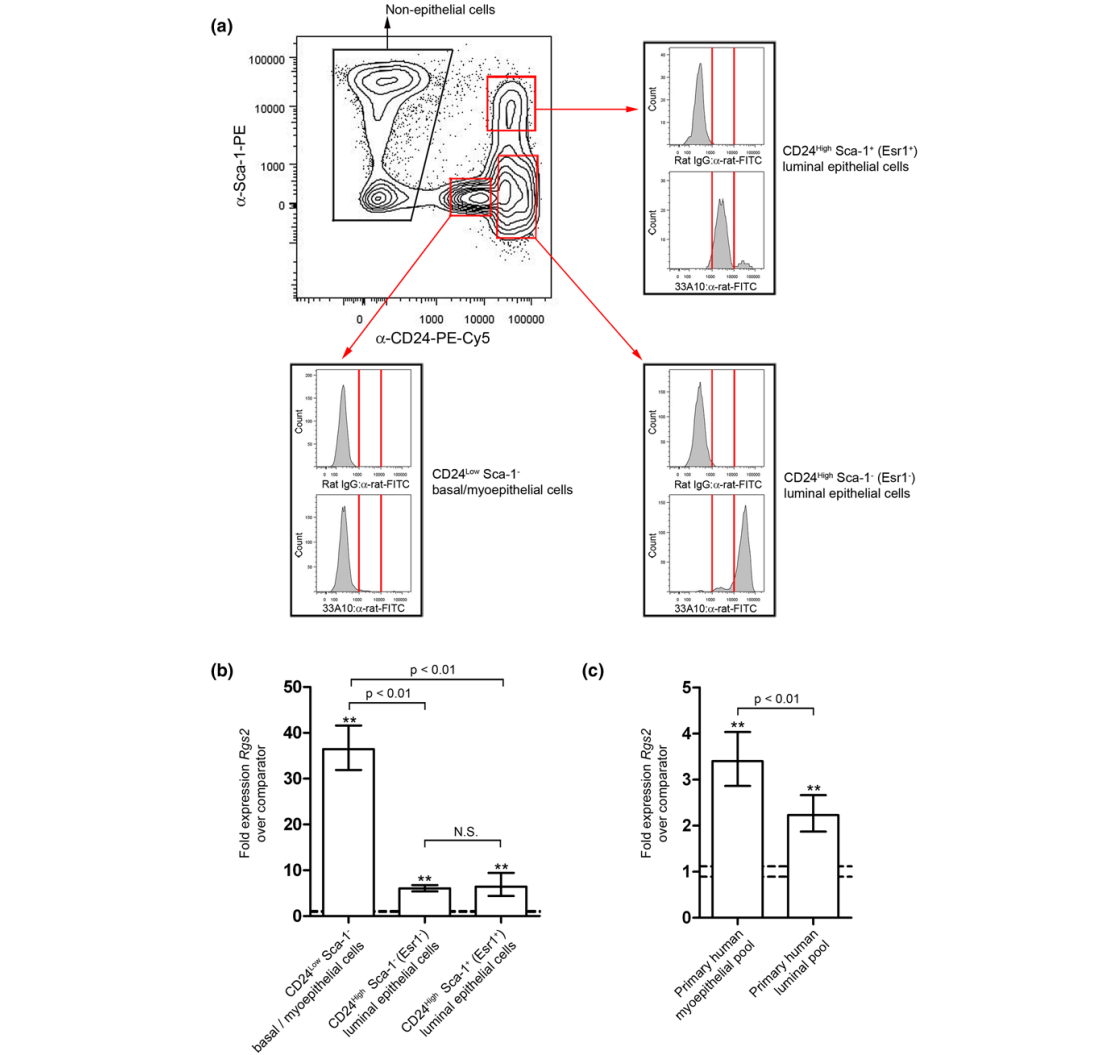
*Rgs2 *is highly expressed in CD24^Low ^Sca-1^- ^33A10^- ^mouse mammary basal/myoepithelial and human breast myoepithelial cells. **(a) **Flow cytometric staining profile of mouse mammary cell preparations stained with anti-Sca-1 and anti-CD24 antibodies together with either a nonspecific rat IgG and anti-rat-FITC or 33A10 and anti-rat FITC. The nonspecific IgG and 33A10 staining profiles of the CD24^Low ^Sca-1^- ^basal/myoepithelial cells (33A10^-^), CD24^High ^Sca-1^- ^(Esr1^-^) luminal cells (33A10^High^) and CD24^High ^Sca-1^+ ^(Esr1^+^) luminal cells (33A10^Low^) [14] are indicated. **(b) **Mean fold differences ± 95% confidence limits in RNA abundance for the *Rgs2 *gene in CD24^Low ^Sca-1^- ^basal/myoepithelial, CD24^High ^Sca-1^- ^(Esr1^-^) luminal epithelial and CD24^High ^Sca-1^+ ^(Esr1^+^) luminal epithelial mouse mammary cells (*n *= 3 for all samples) [14]. The dotted lines indicate the 95% confidence limits of the comparator sample. All samples show a significant difference to the comparator (**P < 0.01). The basal myoepithelial cells also have a significantly higher level of *Rgs2 *expression than either of the two luminal populations. **(c) **Mean fold differences ± 95% confidence limits in expression levels for the *RGS2 *gene in myoepithelial and luminal epithelial human breast cells compared with human breast fibroblasts (comparator). See Materials and methods for details of the samples. The dotted lines indicate the 95% confidence limits of the comparator sample. Both samples show a significant difference to the comparator (**P < 0.01) and the myoepithelial cells have a significantly higher level of *RGS2 *expression than the luminal cells.

To confirm the identity of the Sca-1^- ^33A10^High ^cells, freshly isolated mouse mammary cell preparations were stained with α-CD24, α-Sca-1 and 33A10, and analyzed by flow cytometry. The results (Figure 2a) demonstrated that CD24^Low ^Sca-1^- ^basal/myoepithelial mouse mammary cells were 33A10^- ^and that CD24^High ^Sca-1^- ^(Esr1^-^) luminal cells were 33A10^High^. The data also demonstrated that CD24^High ^Sca-1^+ ^(Esr1^+^) luminal cells did in fact stain with 33A10 but to a lesser extent than the CD24^High ^Sca-1^- ^(Esr1^-^) population, forming a distinct 33A10^Low ^population. This confirmed 33A10 as a marker of all mouse mammary luminal epithelial cells, but it also showed quantitative differences in staining on different luminal epithelial populations. It also confirmed and extended our previous findings that Esr1^+ ^and Esr1^- ^luminal epithelial cells of the mouse mammary gland are distinct epithelial populations.

### *Rgs2 *is highly expressed in the basal/myoepithelial cells of the mammary gland

The gene expression data was next interrogated for genes significantly differentially regulated within the cell populations identified by 33A10 and α-Sca-1 co-staining. We noted that *Rgs2*, which encodes a small GTPase-activating protein (GAP) that is involved in the negative regulation of signalling from G proteins, in particular Gα_q_, was significantly downregulated in the Sca-1^+ ^33A10^Low/- ^and Sca-1^- ^33A10^High ^populations, as well as in fibroblasts. However, it was highly expressed in Sca-1^- ^33A10^Low/- ^cells. As the latter population consisted of both CD24^Low ^basal/myoepithelial cells and nonepithelial cells, but *Rgs2 *was under-expressed in the fibroblasts, we reasoned that it must be strongly expressed specifically in the basal/myoepithelial population. To test this, mouse mammary CD24^Low ^Sca-1^- ^basal/myoepithelial cells, CD24^High ^Sca-1^- ^(Esr1^-^) luminal epithelial cells and CD24^High ^Sca-1^+ ^(Esr1^+^) luminal cells [14] were freshly isolated and qPCR analysis of the levels of *Rgs2 *mRNA was carried out, using mouse mammary fibroblasts as a comparator. The results (Figure 2b) confirmed that *Rgs2 *mRNA expression was strongly upregulated in basal/myoepithelial cells compared with the fibroblast comparator population and both luminal epithelial populations. However, *Rgs2 *expression could still be detected in the luminal epithelial populations.

To determine whether the *RGS2 *expression pattern in human cells was similar to that of the mouse, we analysed gene expression data from three different microarray platforms (Affymetrix, Agilent and Codelink), comparing pooled RNA from primary normal human myoepithelial and luminal epithelial cells (RNA isolated from 10 different preparations in each pool) [29]. The results did not show significant differences between *RGS2 *expression in normal human myoepithelial and luminal cells.

Because this result disagreed with the mouse expression data, we used qPCR to better quantify *RGS2 *expression levels in pooled human normal primary myoepithelial and luminal epithelial cells [29]. Human breast fibroblasts [32] were used as a comparator. This analysis (Figure 2c) demonstrated that, as with the mouse expression data, *RGS2 *expression could be detected in both myoepithelial and luminal epithelial populations. However, although qPCR showed that *RGS2 *expression levels were also significantly higher in the human myoepithelial cells compared with the human luminal cells, the differences were modest compared with those observed with the mouse cells. These modest differences could apparently not be detected by the array platforms. These data did confirm, however, that the pattern of *RGS2 *expression in human and mouse mammary epithelial cells was similar.

### RGS2 is a regulator of oxytocin receptor signalling

RGS2 is a GAP that switches off G-protein activity (particularly Gα_q _activity) and therefore acts as a negative regulator of GPCR signalling [38]. To identify potential functions for RGS2 in the breast, candidate GPCR pathways that might be regulated by RGS2 were examined. One good candidate was the oxytocin receptor pathway. The oxytocin receptor is a GPCR related to the vasopressin receptor, which signals through both Gα_q _and Gα_i _subunits [36], and in the breast it is specifically located in the myoepithelium, the same compartment that is enriched for *RGS2 *expression.

**Figure 3:**
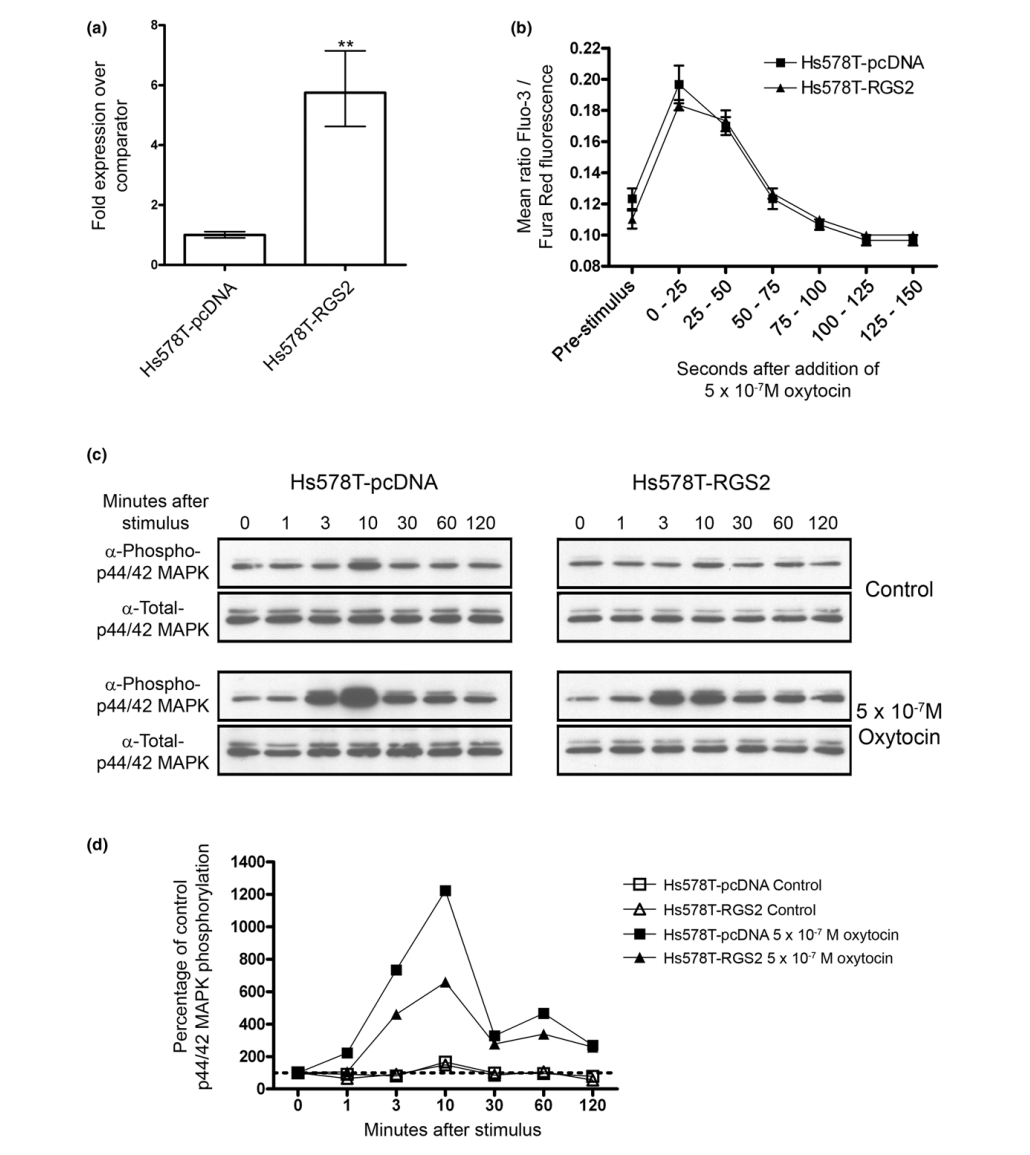
*RGS2 *overexpression attenuates oxytocin receptor signalling to p44/42 MAPK. **(a) **quantitative real-time PCR analysis of *RGS2 *gene expression in triplicate samples of stably transfected Hs578T-pcDNA (parental vector) compared with Hs578T-RGS2 cells. Mean expression levels ± 95% confidence limits are shown. Significant differences are indicated (**P < 0.01). *RGS2 *was fivefold overexpressed in the Hs578T-RGS2 cells compared with the control cell line. **(b) **Analysis of calcium flux in Hs578T-pcDNA and Hs578T-RGS2 cells stimulated with 5 × 10^-7 ^mol/l oxytocin. Both cell lines showed an identical response to the stimulus. **(c) **Time course analysis of p44/42 mitogen-activated protein kinase (MAPK) phosphorylation in Hs578T-pcDNA and Hs578T-RGS2 cells stimulated with 5 × 10^-7 ^mol/l oxytocin. **(d) **Quantitation of phosphorylation analysis. p44/42 MAPK phosphorylation was significantly reduced (*P *< 0.001) in the Hs578T-RGS2 cell line compared with the control Hs578T-pcDNA cells.

To test whether RGS2 was a regulator of oxytocin signalling, the effects of *RGS2 *over-expression were examined in Hs578T cells, which have an endogenous, functional oxytocin receptor [34]. We established a pair of cell lines, one of which stably expressed the empty parental vector (Hs578T-pcDNA) whereas the other stably expressed *RGS2 *(Hs578T-RGS2). qPCR analysis confirmed that the Hs578T-RGS2 line had fivefold *RGS2 *over-expression compared with the control cell line (Figure 3a).

Intracellular signalling downstream from the oxytocin receptor is mediated both by calcium signalling [39] and via p44/42 MAPK [36,40]. To determine whether RGS2-regulated calcium signalling downstream of oxytocin receptor activation, a calcium flux assay was carried out on the Hs578T-pcDNA and Hs578T-RGS2 cells. The results (Figure 3b) showed that the calcium flux in these cells following oxytocin stimulation was identical, indicating that RGS2 does not regulate this pathway. Next, p44/42 MAPK phosphorylation in response to oxytocin stimulation in the cell lines was examined. Cells were serum starved and insulin starved for 2 hours, stimulated with 5 × 10^-7 ^mol/l oxytocin and then harvested at different time points for assessment of levels of phosphorylated, active p44/42 MAPK (Figure 3c,d). In two independent experiments, *RGS2 *over-expression significantly (*P *< 0.001 in both experiments) reduced p44/42 MAPK phosphorylation after oxytocin stimulation.

**Figure 4:**
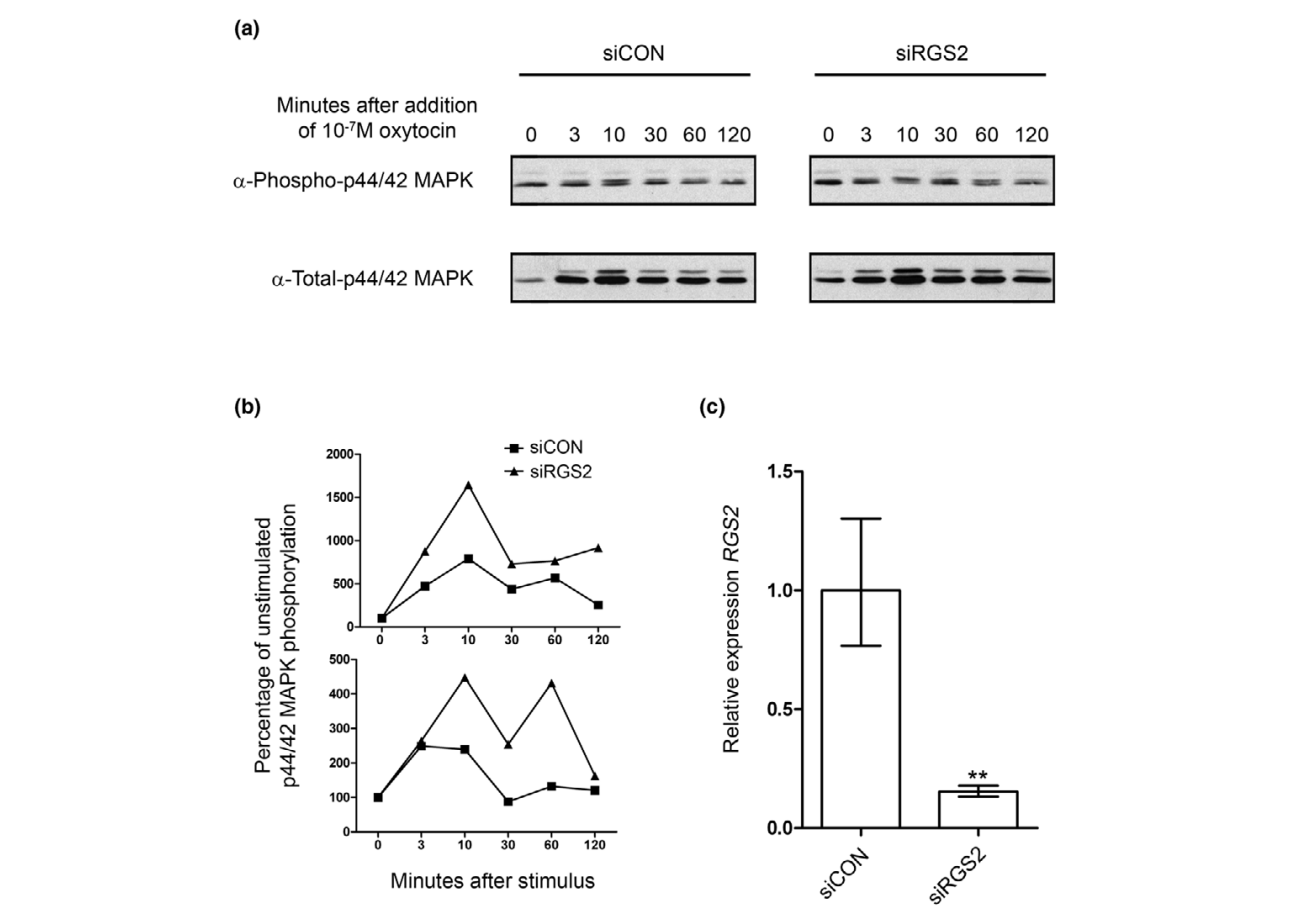
Silencing of endogenous *RGS2 *enhances oxytocin receptor signalling via p44/42 MAPK in Hs578T cells. **(a) **Hs578T cells transfected with either a scrambled control small interfering (si)RNA (SiCON) or an siRNA targeting *RGS2 *(siRGS) were serum and insulin starved for 2 hours then stimulated with 1 × 10^-7 ^mol/l oxytocin. Cells were harvested at timepoints up to 120 minutes after oxytocin addition and lysates analysed for levels of phospho- and total p44/42 mitogen-activated protein kinase (MAPK) by immunoblotting. **(b) **Quantitation of siRNA knockdown from two independent transfections. The upper plot is the quantitation of the blots shown in panel a. siRGS2 caused a significant (*P *< 0.001) increase in phosphorylation in response to oxytocin. **(c) **Quantitative real-time PCR analysis of *RGS2 *expression in Hs578T cells transfected with the siRGS2. Each bar represents the mean ± 95% confidence limits of the fold difference in expression compared with the mean expression in the siCON transfected cells. Data from four samples harvested from two independent transfections is shown (one of which was also used in the lower oxytocin response experiment shown in panel b). Significant differences are indicated (**P < 0.01).

To confirm this result, we examined the effects on p44/42 MAPK phosphorylation of silencing endogenous *RGS2 *in wild-type Hs578T cells. Cells transfected with either a control (siCON) siRNA or an siRNA targeting *RGS2 *(siRGS2) were serum and insulin starved and then treated with 1 × 10^-7 ^mol/l oxytocin. A lower concentration of oxytocin was used to give a reduced stimulation, which could be measurably altered by *RGS2 *silencing. Again, cells were harvested at different time points and assayed for levels of phospho-p44/42 MAPK. In two independent experiments, silencing of endogenous *RGS2 *in wild-type Hs578T cells caused a significant (*P *< 0.001) increase in p44/42 MAPK phosphorylation following oxytocin stimulation (Figure 4a,b). Efficient silencing of endogenous *RGS2 *was confirmed by qPCR (Figure 4c).

### *RGS2 *is over-expressed in the majority of breast cancers

Finally, we examined the pattern of expression of *RGS2 *in human breast cancer. A data mining approach was first used to examine *RGS2 *expression in a set of gene expression data from normal breast samples (10 pooled isolates of freshly separated normal luminal cells) and breast cancers (15 freshly isolated infiltrating ductal carcinomas of grade 2 or 3, from which cells expressing the desmosplastic fibroblast antigen F19 had been removed) analyzed by four different gene expression microarray platforms and massively parallel signature sequencing [29]. The data from all four microarray platforms showed that *RGS2 *was significantly upregulated in malignant breast cancers compared with normal luminal cells (Affymetrix: 3.2-fold increase, *P *= 3.77 × 10^-10^; Agilent: 2.49-fold increase, *P *= 1.25 × 10^-6^; CodeLink: 3.02-fold increase, *P *= 2.14 × 10^-10^; brk IMAGE: 2.71-fold increase, *P *= 1.35 × 10^-6^). The massively parallel signature sequencing analysis quantified the tumour pool as having 96 *RGS2 *transcripts per million and the normal luminal pool as having 0 transcripts per million (the gene was not detected in two independent sequencing runs).

We next compared expression of *RGS2 *in the tumour pool with the pooled normal human myoepithelial cells on the Affymetrix, Agilent and Codelink platforms. These data showed that the RGS2 expression was also significantly upregulated in the tumour pool compared with the normal myoepithelial pool (Affymetrix: 3.48-fold increase, *P *= 2.23 × 10^-10^; Agilent: 2.85-fold increase, *P *= 4.71 × 10^-5^; CodeLink: 12.9-fold increase, *P *= 1.14 × 10^-4^).

**Figure 5:**
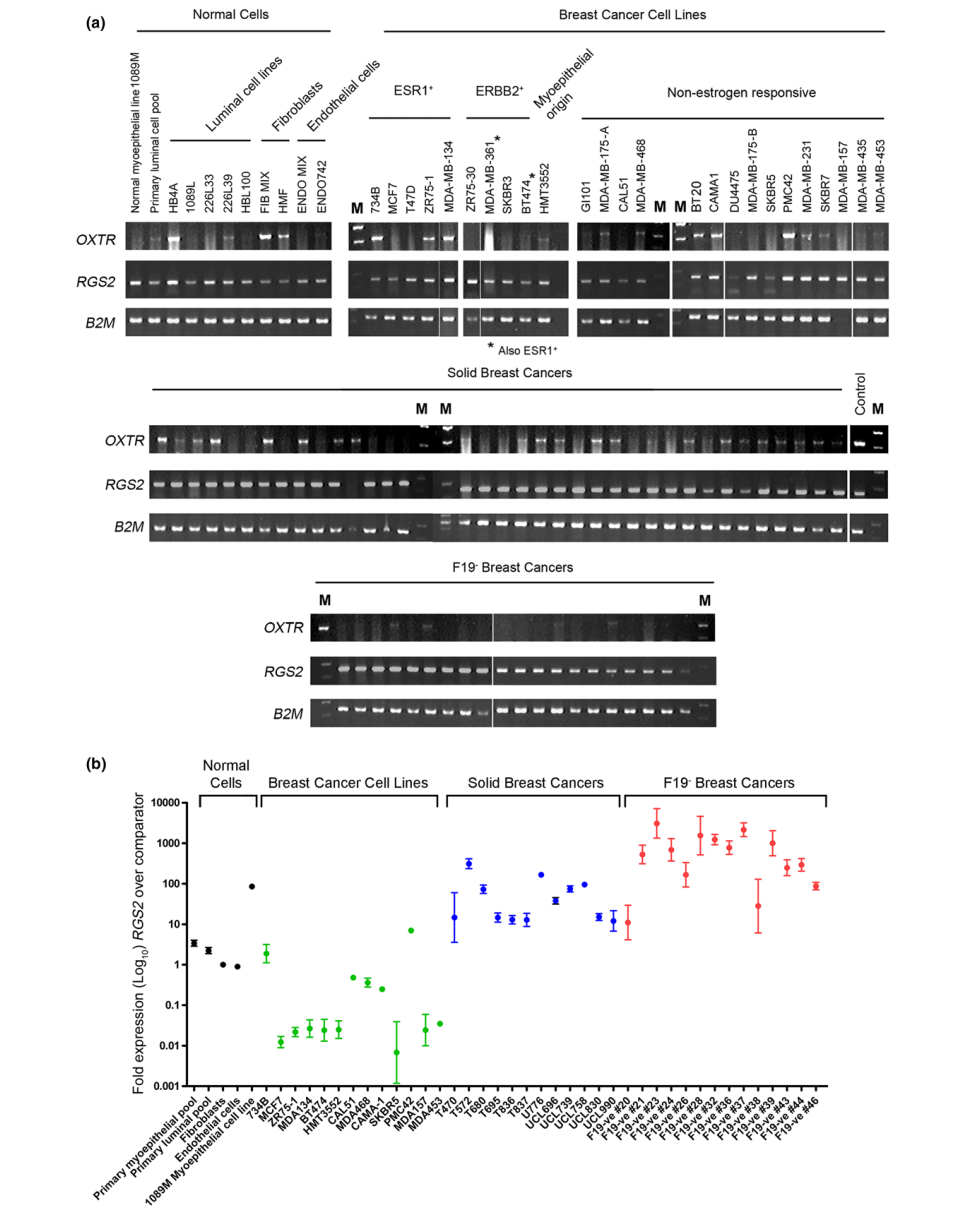
RGS2 is expressed in human myoepithelial and luminal cells and in breast cancers. **(a) **RNA isolated from normal primary breast cells, normal and breast cancer cell lines and primary breast cancers was analysed by semi-quantitative RT-PCR for expression at the transcriptional level of *RGS2, OXR *and a housekeeping gene *B2M*. **(b) **Quantitative real-time PCR analysis of RGS2 expression levels in a selection of primary human cells, human breast cancer cell lines, solid breast cancers and F19-depleted cancers. Data are mean relative expression levels ± 95% confidence limits (*n *= 3 analyses of each sample). For comparison, the primary myoepithelial and primary luminal cell data from Figure 2c have been included on this graph. Note that the y-axis is a log10 scale.

To validate these findings, a panel of samples including normal breast cells, breast cancer cell lines (including ESR1^+ ^cell lines, estrogen nonresponsive cell lines, ERBB2 over-expressing cell lines and 55 primary tumour samples, some of which had been depleted of F19-expressing fibroblasts) were analysed by semi-quantitative RT-PCR for *RGS2 *expression. The samples were also analysed for *OXTR *expression levels. The results (Figure 5a) confirmed the *in silico *analysis, with *RGS2 *expression seen in the majority of samples, being particularly strong in the solid tumour and F19-depleted samples. In contrast, *OXTR *expression in cell lines and primary tumours was variable, with only two out of four ESR1^+ ^breast cancer cell lines, one of three ERBB2 over-expressing cell lines, one basal origin tumour cell line, eight out of 17 (47%) estrogen nonresponsive cell lines, and 23 out of 56 (41%) primary tumours having detectable oxytocin receptor gene expression.

For more accurate quantitation of levels of *RGS2 *mRNA expression in cell lines and primary tumours, a selection of cell lines, solid tumours and F19-depleted tumours was examined by qPCR. The results (Figure 5b) showed that compared with normal primary cells (either myoepithelial or luminal epithelial cells, breast fibroblasts, or endothelial cells) most cell lines had a much reduced level of *RGS2 *expression. One exception was the 1089M immortalized normal myoepithelial cell line, which had high levels of *RGS2 *expression. However, *RGS2 *expression was significantly increased in the solid tumours compared with the normal primary cells. It was increased still further in the F19-depleted cancers compared with all samples (including the 1089M cell line), some of which had levels of *RGS2 *expression 1,000-fold or more times that of the comparator. This suggests that *RGS2 *mRNA expression is strongly upregulated specifically in the epithelial cells of the majority of primary breast cancers.

## Discussion

In our previous studies, we found that the mouse mammary epithelium could be separated into total luminal or myoepithelial fractions by using the 33A10 and JB6 antibodies respectively [17,18], and, more recently, that anti-CD24 staining identifies three distinct mammary cell populations, namely CD24^- ^nonepithelial cells, CD24^Low ^basal/myoepithelial cells and CD24^High ^total luminal epithelial cells [12]. The CD24^High ^total luminal fraction could be further subdivided into Sca-1^+ ^Prominin^+ ^Esr1^+ ^and Sca-1^- ^Prominin-1^- ^Esr1^- ^fractions [14]. In the present study we expected that, because the mouse mammary luminal fraction can be isolated with both 33A10 and anti-CD24 staining, both the CD24^High ^Sca-1^+ ^and CD24^High ^Sca-1^- ^cells would have identical 33A10 staining patterns. Instead, we found that the 33A10 antibody differentially stained the luminal epithelial populations. We were able initially to identify the Sca-1^- ^33A10^High ^population as being identical to CD24^High ^Sca-1^- ^cells on the basis of patterns of gene expression. Subsequent flow cytometric analysis confirmed that 33A10 staining differentiated between the two luminal epithelial populations we previously identified [14]. CD24^High ^Sca-1^- ^(Esr1^-^) luminal epithelial cells had a 33A10^High ^staining pattern whereas CD24^High ^Sca-1^+ ^(Esr1^+^) luminal epithelial cells were 33A10^Low^. As expected, CD24^Low ^Sca-1^- ^basal/myoepithelial cells were 33A10^-^. This new differential staining pattern of the 33A10 antibody was most likely observed because of advances in flow cytometry technology and analysis software since our early observations, although mouse strain differences in the pattern of expression of the antigen recognized by the 33A10 antibody (most likely a milk fat globule membrane antigen) [15,16] cannot be excluded.

With these data, we were able to extend our analysis of the mouse mammary epithelium, confirming that there are two distinct populations of luminal epithelial cells [14]. Furthermore, we used the gene expression array data that formed the basis for defining the identity of the Sca-1^- ^33A10^High ^population to identify *Rgs2 *as a gene highly differentially regulated between the epithelial populations of the mammary gland. Remarkably, *RGS2 *is over-expressed in the majority of breast cancer cell lines and primary breast tumours. RGS2 and the pathways it regulates are therefore important new candidate therapeutic targets for the treatment of breast cancer.

RGS2 is a regulator of GPCRs, which acts as a GTPase-activating protein (GAP), primarily (although not exclusively) downregulating signalling from the Gα_q _subunit [41]. GPCRs are the largest family of cell-surface molecules involved in signal transduction [42]. They all have a core of seven transmembrane α-helices and, after agonist binding, interact with the αβγ G-protein heterotrimers. This catalyzes the dissociation of Gα-bound GDP and its replacement with GTP, leading to dissociation of the Gβγ from the Gα subunits. The Gα• GTP and Gβγ subunit complexes then both stimulate downstream effectors until the GTP is hydrolyzed back to GDP and the signal switched off [42]. GTP hydrolysis is regulated by the RGS proteins, which act as GAPs for Gα subunits, although they can also have GAP-independent regulatory functions [43].

The diverse signalling roles of GPCRs in cancer were recently reviewed [42]. In breast cancer, these include potential roles in growth, metastasis, angiogenesis and hormone therapy resistance. Given this diversity, determination of the specific function(s) of RGS2 in the context of the breast and breast cancer is extremely difficult. *Rgs2 *knockout mice were viable and had normal reproduction, although mammary phenotypes and lactation were not examined [41,44]. The mice did, however, exhibit a hypertensive phenotype, with renovascular abnormalities, persistent constriction of resistance vasculature and prolonged response of vasculature to vasoconstrictors. These effects were mediated through angiotensin II signalling and α_1_-adrenergic receptors and the hypertensive phenotype could be blocked with AT1 receptor antagonists [41]. Analysis of the mice also demonstrated that Rgs2 plays role in T-cell activation, synapse development in hippocampus and emotive behaviours [44]. The involvement of the gene in mediating vascular constriction and its strong over-expression in the basal mammary cell compartment suggested that at least one role for RGS2 in the breast is as a regulator of oxytocin signalling in the mammary gland, regulating myoepithelial cell contraction during lactation. Indeed, the data presented here confirm that RGS2 modulates signalling of the oxytocin receptor to p44/42 MAPK but not oxytocin receptor-mediated calcium signalling, suggesting that it does indeed regulate signalling of this receptor in the myoepithelial cells.

Remarkably, *RGS2 *gene expression was also upregulated in the majority of breast cancers examined, suggesting that dysfunction in the pathway or pathways regulated by RGS2 in the breast is an obligate event in breast cancer development. *RGS2 *and *OXTR *expression levels were not correlated in breast cancer, suggesting that *RGS2 *modulates a different GPCR pathway(s) in breast tumours. Exactly which pathway remains unclear. A number of GPCRs have been previously reported to be overexpressed in breast cancer, including CCR1, Opsin-3, CXCR4, PAR1, PAR2, EP2, EP4, GPR30 and the C3A anaphylatoxin chemotactic receptor [42,45], although whether any of these are regulated by RGS2 remains unknown. One clue to the pathways involved may be that RGS2 was upregulated in the solid breast cancers, and in particular in the F19-depleted cancers, but tended to be less strongly expressed in cancer cell lines than in the normal tissue. This suggests that *RGS2 *expression in tumours is a consequence of interactions with the tumour microenvironment.

Identification of the GPCR pathway(s) regulated by RGS2 in breast cancer will be key to assessing whether it has a critical function in cancer biology and whether this pathway is of value as a therapeutic target. GPCRs can have tumour suppressor activity, as has been noted for GPR54 and metastasis in breast cancer [42], and tumour development would lead to selective pressure to block such pathways. Mechanisms for blocking the activity of tumour suppressor GPCR pathways could include upregulation of negative regulators of GPCRs, such as RGS2. Alternatively, GPCRs can provide cellular proliferation and survival signals [42,46,47], possibly in response to interactions with the tumour microenvironment, and RGS2 upregulation may be the result of a negative feedback mechanism. This would be consistent with its high expression in *in situ *cancers (Figure 5b). In this case, alternative survival signals might be selected for in tumour development to bypass the RGS2 block. Identifying and blocking alternative survival signals may enable a 'synthetic lethality' approach [48] to treating breast cancers with high levels of RGS2. Such an approach is attractive because direct targeting of RGS2 itself may prove problematic because of possible side effects resulting from its role in the regulation of vasoconstriction. However, the very large difference in *RGS2 *expression between F19-depleted tumours and normal endothelial cells (Figure 5b) suggests that a pronounced therapeutic window for RGS2 targeting exists.

Activity of p44/42 MAPK tends to be high in breast cancer [49], and so it is perhaps counterintuitive that RGS2, which suppresses p44/42 MAPK phosphorylation in oxytocin signalling, is highly expressed in breast cancers and cancer cell lines. However, RGS2 acts at the level of the G protein in the signalling pathway, not on p44/42 MAPK itself. RGS2 could suppress GPCR signalling even if p44/42 MAPK is activated via alternative routes. Furthermore, intracellular signalling is compartmentalized by spatial restriction mechanisms such as immobilization in lipid rafts [50,51] or attachment to scaffold proteins [52,53], and RGS2 could block phosphorylation of the GPCR signalling-associated p44/42 MAPK pool without affecting other pools. Thus p44/42 MAPK activity could be high in a tumour, even if the GPCR-associated activity is low.

## Conclusion

We have confirmed and extended our previous analyses of prospectively isolated mouse mammary epithelial subpopulations by demonstrating that different levels of 33A10 antibody staining identify different luminal epithelial subpopulations. Furthermore, molecular analysis of prospectively isolated mammary epithelial cells has identified Rgs2 as a modulator of oxytocin receptor signalling that is expressed most strongly in the myoepithelial cell layer. The *RGS2 *gene, but not the *OXTR *gene, was also shown to be over-expressed in the majority of breast cancers, identifying the product of this gene, or the pathway(s) it regulates, as potentially significant therapeutic targets. However, because the function of RGS2 is unlikely to be mediated through oxytocin receptor signalling in breast cancer, its role in the pathogenesis of the disease remains to be elucidated.

## Abbreviations

EGFR = epidermal growth factor receptor; FCS = foetal calf serum; GAP = GTPase-activating protein; GPCR = G-protein coupled receptor; HER = human epidermal growth factor receptor; MAPK = mitogen-activated protein kinase; PBS = phosphate-buffered saline; qPCR = quantitative real time PCR; RGS2 = regulator of G-protein signalling 2; RT-PCR = reverse transcription polymerase chain reaction; SSC = sodium chloride/citrate.

## Competing interests

The authors declare that they have no completing interests.

## Authors' contributions

MJS designed the study, isolated mouse mammary cell populations, conducted biochemical analysis of RGS2 function, analyzed and interpreted the data, and drafted the manuscript. MI performed the microarray experiments and assisted with data analysis. ML performed and analysed the RT-PCR experiments on tumour samples. AG and TD carried out the bioinformatics analysis of the microarray data. HK and JLR performed the quantitative RT-PCR experiments and assisted with data analysis. KF and AM generated the in-house microarrays and assisted with data analysis. KB harvested mouse mammary fibroblasts and assisted with data analysis. SM and CJL helped design, carried out and interpreted siRNA knockdown assays. AA helped design the study and interpret the data and helped draft and revise the manuscript. All authors assisted with drafting the manuscript and approved the final version.

## Supplementary Material

Additional file 1 An .XLS file showing the results of cDNA microarray analysis of Sca-1^+^/33A10^Low/- ^mammary cells with reference population. Genes are ranked by their M ratio. Only well annotated genes with a significant *P *value (<0.05) are shown. SOURCE Clone ID [54], UniGene ID, gene name and gene symbol are also given for each gene. Click here for file

Additional file 2 An .XLS file showing the results of cDNA microarray analysis of Sca-1^-^/33A10^High ^mammary cells with reference population. Genes are ranked by their M ratio. Only well annotated genes with a significant *P *value (<0.05) are shown. SOURCE Clone ID [54], UniGene ID, gene name and gene symbol are also given for each gene. Click here for file

Additional file 3 An .XLS file showing the results of cDNA microarray analysis of Sca-1^-^/33A10^Low/- ^mammary cells with reference population. Genes are ranked by their M ratio. Only well annotated genes with a significant *P *value (<0.05) are shown. SOURCE Clone ID [54], UniGene ID, gene name and gene symbol are also given for each gene. Click here for file

Additional file 4 An .XLS file showing the results of cDNA microarray analysis of mammary fibroblasts with reference population. Genes are ranked by their M ratio. Only well annotated genes with a significant *P *value (<0.05) are shown. SOURCE Clone ID [54], UniGene ID, gene name and gene symbol are also given for each gene. Click here for file

Additional file 5 An .XLS file showing the comparison of cDNA microarray data of all significant genes across all cell populations. Genes are listed alphabetically and their M ratio and *P *value (<0.05) for each cell population are shown. SOURCE Clone ID [54], UniGene ID, gene name and gene symbol are also given for each gene. Click here for file

Additional file 6 A .TIFF file showing the Gene Ontology biological process analysis of microarray data. Presented is a graphical representation of percentages of genes involved in differing biological processes, as defined by Gene Ontology annotation, for each cell population. Click here for file
