# Supplementary figures and images for: Regulator of G-protein signalling 2 mRNA is differentially expressed in mammary epithelial subpopulations and over-expressed in the majority of breast cancers
Source: Breast Cancer Res. 2007 Dec 8;9(6):R85. doi: 10.1186/bcr1834 (PMC2246188; doi:10.1186/bcr1834)

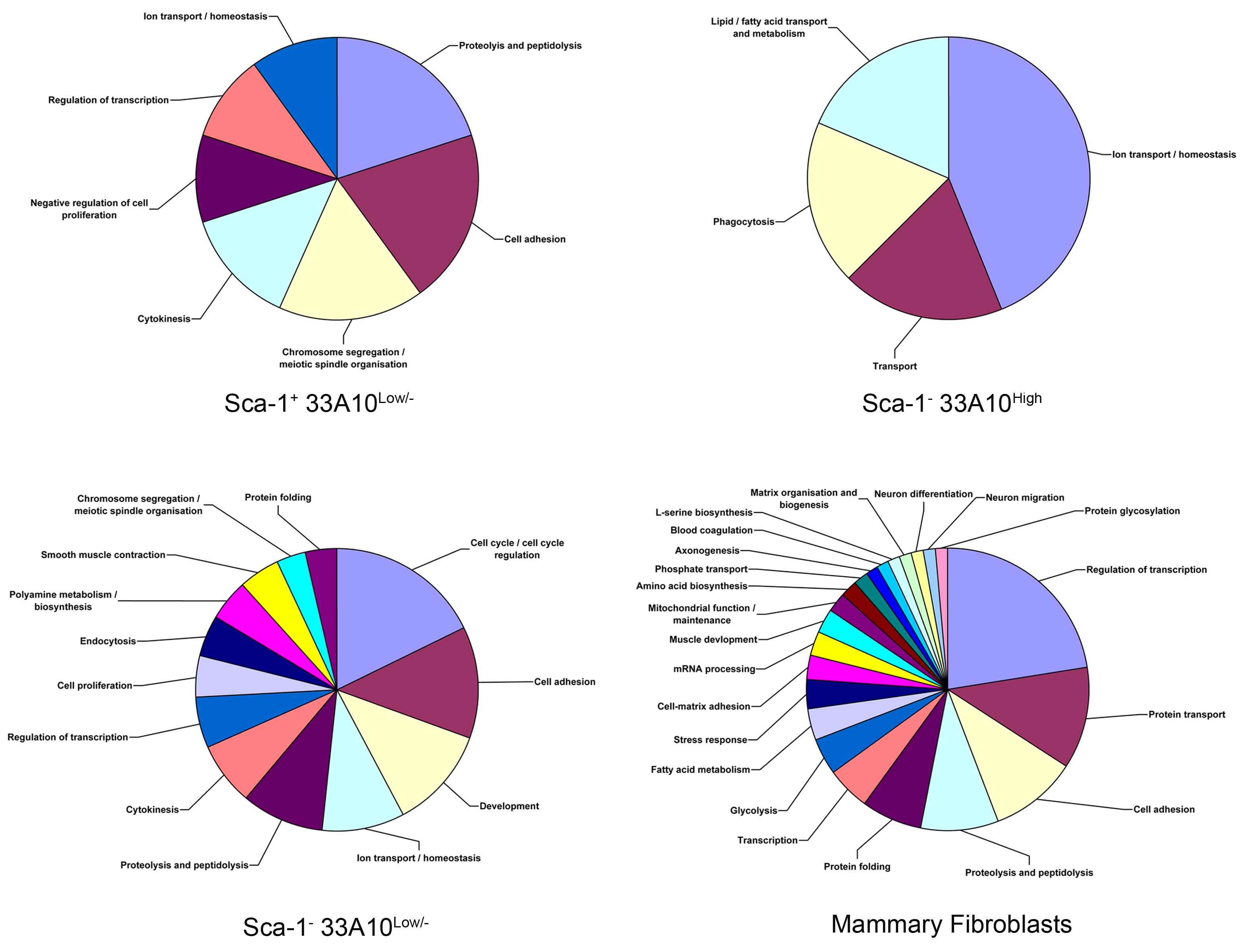

Supplement: Additional file 6 — A .TIFF file showing the Gene Ontology biological process analysis of microarray data. Presented is a graphical representation of percentages of genes involved in differing biological processes, as defined by Gene Ontology annotation, for each cell population. [file bcr1834-S6.tiff]
